# The Golgi as a “Proton Sink” in Cancer

**DOI:** 10.3389/fcell.2021.664295

**Published:** 2021-05-13

**Authors:** Koen M. O. Galenkamp, Cosimo Commisso

**Affiliations:** Cell and Molecular Biology of Cancer Program, NCI-Designated Cancer Center, Sanford Burnham Prebys Medical Discovery Institute, La Jolla, CA, United States

**Keywords:** Golgi pH, proton sink, pH homeostasis, ion transport, intracellular pH, cancer

## Abstract

Cancer cells exhibit increased glycolytic flux and adenosine triphosphate (ATP) hydrolysis. These processes increase the acidic burden on the cells through the production of lactate and protons. Nonetheless, cancer cells can maintain an alkaline intracellular pH (pHi) relative to untransformed cells, which sets the stage for optimal functioning of glycolytic enzymes, evasion of cell death, and increased proliferation and motility. Upregulation of plasma membrane transporters allows for H^+^ and lactate efflux; however, recent evidence suggests that the acidification of organelles can contribute to maintenance of an alkaline cytosol in cancer cells by siphoning off protons, thereby supporting tumor growth. The Golgi is such an acidic organelle, with resting pH ranging from 6.0 to 6.7. Here, we posit that the Golgi represents a “proton sink” in cancer and delineate the proton channels involved in Golgi acidification and the ion channels that influence this process. Furthermore, we discuss ion channel regulators that can affect Golgi pH and Golgi-dependent processes that may contribute to pHi homeostasis in cancer.

## Introduction

**FIGURE 1:**
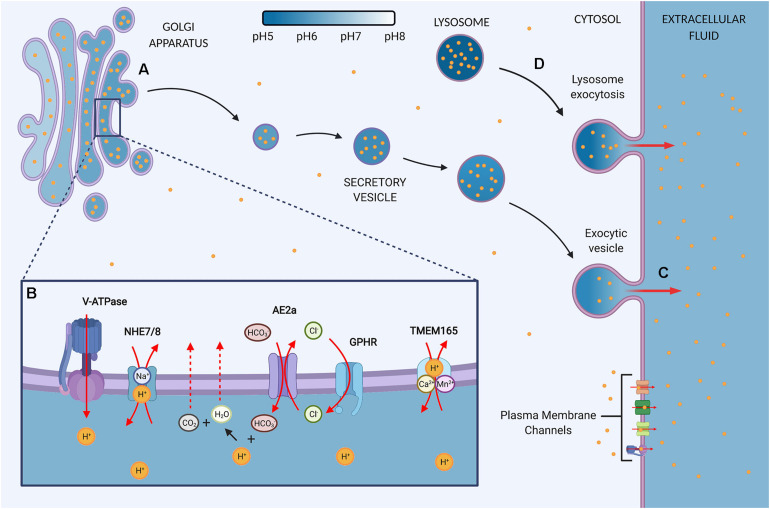
The Golgi contains proton channels and inherent properties that may convert the organelle into a proton sink in cancer. **(A)** The Golgi is an acidic organelle that shows a decreasing pH gradient along the sub-compartments, eventually leading to the formation of acidic secretory vesicles and granules. The luminal proton concentration is 10–100 times higher as the cytosol and thus the organelle may function as a proton repository that contributes to the upkeep of an alkaline intracellular pH (pHi) in cancer cells. The resting pH of the Golgi and vesicles is thought to be mediated by proton loading and counter ion conductance. Additionally, a proton leak pathway allows for reducing luminal proton content, but the pathway is suggested to be absent in secretory vesicles. **(B)** Ion channels at the Golgi regulate the luminal H^+^ content. V-ATPase: Vacuolar H^+^-ATPases load the lumen constitutively with protons in an ATP-dependent manner. NHE7/8: Sodium-hydrogen exchanger 7 (NHE7) has been implicated in proton loading by exchanging protons for luminal Na^+^. However, the directionality of NHEs at the Golgi is debated since other studies propose that NHE7 and NHE8 function as a proton leak pathway. AE2a: Anion exchanger 2a is a Golgi-residing AE2 isoform that buffers the Golgi through HCO_3_^–^ loading in exchange for Cl^–^. The buffering presents a sought-after proton leak pathway by providing means to neutralize luminal protons through the production of water and carbon dioxide, which can exit the lumen through diffusion. The directionality of AE2 is reversible and therefore bicarbonate influx is gradient-dependent. GPHR: Golgi pH regulator loads the lumen with Cl^–^ in a voltage-gated manner. Hence, it provides the chloride ions required for counterion conductance to sustain the constitutive activity of V-ATPases. In addition, GHPR is thought to provide the chloride ions to allow for AE2a-mediated HCO_3_^–^ buffering. TMEM165: Transmembrane protein 165 ion selectivity and directionality is still under investigation but data points toward Ca^2+^/Mn^2+^ transport in exchange for H^+^. **(C)** Secretory vesicles are targeted for exocytosis and thus present a pathway by which the Golgi may target protons to the extracellular space and convert the Golgi into a proton sink by siphoning off cytosolic H^+^. Secretion is upregulated in cancer, but the role in regulating pHi homeostasis remains to be determined. **(D)** In response to an acidic extracellular environment, lysosomes have been shown to be targeted for exocytosis, thereby maintaining an alkaline pHi and protecting the cells from extracellular acid.

Cancer cells require large amounts of energy in the form of adenosine triphosphate (ATP) to drive rapid proliferation and support cellular processes including the activation of cell signaling pathways, membrane transport, and DNA and protein synthesis (Alberts, 2015; Zhu and Thompson, 2019). Interestingly, cancer cells frequently switch to a less efficient glucose metabolism pathway for ATP production, compared to untransformed cells (Heiden et al., 2009; Liberti and Locasale, 2016). This cancer hallmark phenomenon is known as the Warburg effect. Untransformed cells mostly rely on mitochondrial oxidative phosphorylation (OXPHOS) for energy production, which is a highly efficient process where oxygen and glucose-derived pyruvate fuel the TCA cycle and drive the electron transport chain to produce 36 ATPs per glucose molecule (Heiden et al., 2009; Liberti and Locasale, 2016). Although cancer cells are generally equipped with a functional OXPHOS pathway, glucose metabolism is frequently switched to aerobic glycolysis where pyruvate is fermented to lactate, even when oxygen is available. This switch in metabolism is thought to provide essential building blocks for the rapid production of biomass and to facilitate rapid proliferation. However, the pathway leads to the production of only two ATPs per glucose molecule and thus requires the cells to boost glucose consumption to meet energy demands. A byproduct of this metabolic rewiring is the increased production of lactate and a surge in H^+^. Further contributing to the surge in H^+^ is the hydrolysis of ATP that supports cellular processes and accelerated proliferation. High lactate and H^+^ levels can have devastating effects on cellular fitness by lowering the intracellular pH (pHi). Surprisingly though, the pHi of cancer cells is not acidic, but is even more alkaline relative to untransformed cells (Webb et al., 2011; Corbet and Feron, 2017; Zheng et al., 2020). The maintenance of this alkaline pHi provides cancer cells with an optimal environment for glycolytic enzyme activity and cellular advantages to proliferate, migrate, and withstand cell death cues. To avoid the accumulation of cytosolic H^+^, cancer cells express multiple families of transporters on the plasma membrane, including vacuolar H^+^-ATPases, sodium-hydrogen exchangers, and monocarboxylate transporters, all of which can extrude protons (Figure 1). However, increasing evidence shows that the regulation of pH homeostasis in cancer is not as straightforward as extrusion of protons at the plasma membrane level, as acidification of organelles, such as lysosomes and the Golgi, contributes to the maintenance of an alkaline cytosol (Liu et al., 2018; Funato et al., 2020; Galenkamp et al., 2020). These organelles therefore function as repositories for H^+^ storage or means to extrude protons through an alternative pathway. Hence, because of their role in siphoning off cytosolic protons, these organelles can be considered the “proton sinks” of the cell.

## Proton Sinks in Cancer

The presence of acidic organelles and proton pumping into their lumen can conceptually contribute to the upkeep of an alkaline cytosol by sequestering protons or targeting them for the extracellular space through exocytosis. While it is conceivable that both normal and cancer cells might exploit organelle-mediated sequestering of protons for pHi maintenance, the available literature specifically points to this process as being selective to cancer cells (Liu et al., 2018; Funato et al., 2020; Galenkamp et al., 2020). This idea is consistent with the notion that cancer cells produce more acidic moieties relative to untransformed cells due to their altered metabolism.

Acidic organelles in mammalian cells belong to the endocytic and secretory pathway and each of these organelles has a distinct resting pH (Paroutis et al., 2004; Casey et al., 2010). Notably, the Golgi pH gradually descends through the sub-compartments; starting at pH 6.7 at the *cis*-Golgi, reaching pH 6.0 at the *trans-*Golgi network and ultimately ending in the formation of secretory vesicles and granules which can reach pH values as low as 5.2. Lysosomes are the cell’s most acidic organelle with a resting pH that ranges between pH 4.7 and 5.5, and endosomes acidify as they mature; early endosomes have a resting pH of 6.3–6.5 that decreases to pH 5.5 in late endosomes. The luminal pH of these organelles is required for proper organelle function and regulation of the organelle pH is important for maintaining cellular fitness. For instance, in the Golgi, luminal pH levels regulate the activity of glycosyltransferases and vesicular trafficking (Axelsson et al., 2001; Kornak et al., 2008; Maeda et al., 2008; Hucthagowder et al., 2009; Rivinoja et al., 2009), while in the lysosomes the pH activates hydrolases and mediates cargo degradation (Chen et al., 2020).

The pH scale is a negative logarithm and thus these organelles contain proton concentrations that are 10–1,000 times higher than those found in the cytosol of cancer cells, which has pH values of 7.4 and higher (Webb et al., 2011, 2021; Corbet and Feron, 2017; Zheng et al., 2020). Lysosomes, endosomes, and the Golgi each occupy 3% or less of the total volume of a cell (Alberts, 2015; Valm et al., 2017). But due to these high luminal proton concentrations, these organelles may function as proton repositories that contribute to maintaining an alkaline pHi in cancer. Even small changes in the pH of these organelles can translate to high molar quantities of H^+^ ions at the cytosolic level. For instance, in cervical cancer, with lysosomes showing a resting pH of 4.6, an increase of ∼0.7 pH units in the lysosome pH was able to bring about a ∼0.4 pH unit decrease in the cytosol (Liu et al., 2018). Moreover, in pancreatic cancer, a ∼0.5 pH unit increase in *trans-*Golgi network pH resulted in a ∼0.5 pH unit decrease in pHi and ablation of the Golgi through Brefeldin A administration produced similar effects on pHi homeostasis (Galenkamp et al., 2020). In these cases of induced pH alteration of the lysosome and Golgi, the increase in organelle pH and the concomitant decrease in cytosolic pH reduced the viability of the cells, marking the importance of these organelles in maintaining an alkaline pHi and cell fitness in cancer cells.

In addition to storing protons, the Golgi and lysosomes can potentially target protons to the extracellular space through exocytosis, which adds an extra layer by which these organelles can regulate pHi homeostasis (Figure 1; Jaiswal et al., 2009; Rivera-Molina and Toomre, 2013; Deng et al., 2016; Nugues et al., 2018; Funato et al., 2020). Interestingly, exocytosis may be further stimulated in cancer cells by the acidification of the extracellular surroundings and alkalinization of the cytosol. During tumor acidosis, the extracellular fluid can reach pH 6.5 through increased proton and lactate extrusion (Webb et al., 2011; Corbet and Feron, 2017; Zheng et al., 2020). As an adaptation to this acidic environment, lysosomes are targeted for fusion with the plasma membrane to protect the cells from acidity, but thereby also contribute to maintaining an alkaline cytosol (Steffan et al., 2009; Damaghi et al., 2015; Funato et al., 2020). Moreover, cytosolic acidification was discovered to inhibit Golgi to plasma membrane trafficking (Cosson et al., 1989), while an alkaline pHi promotes exocytosis (Pernas-Sueiras et al., 2005; Huck et al., 2007). An alkaline cytosol was recently shown to change the protonation status of PI4P, a phosphatidylinositol that predominantly localizes to the Golgi and is required for secretory vesicle formation (Dippold et al., 2009; Rahajeng et al., 2019; Waugh, 2019; Shin et al., 2020). Using a yeast model system, it was demonstrated that reduced PI4P protonation in response to cytosolic alkalinization increased PI4P protein binding (Shin et al., 2020). It remains to be determined whether this protonation status promotes secretory vesicle formation. Nonetheless, PI4P is an important docking station for GOLPH3, a Golgi-localizing oncoprotein frequently upregulated in cancer, which is described to increase secretory vesicle formation and Golgi-to-plasma membrane trafficking (Halberg et al., 2016; Waugh, 2019; Sechi et al., 2020). Multiple cancer types have been found to display increased and malignant Golgi-dependent secretion that drives metastasis, a process enhanced by extracellular acidification (Webb et al., 2011; Halberg et al., 2016; Corbet and Feron, 2017; Capaci et al., 2020; Gupta et al., 2020; Tan et al., 2020; Zheng et al., 2020). Whether the malignant secretion presents a pathway to boost proton extrusion through the Golgi and whether that contributes to the metastatic potential of cancer cells and pHi homeostasis is a concept that requires further investigation (Figure 1).

Organelle pH levels are tightly regulated to support cell function; however, changes in organelle pH are detected in cancer. Multiple cancer types display a decreased lysosome pH compared to tissue-matched untransformed cells (Webb et al., 2021). Lysosomes of triple negative breast cancer cells are significantly more acidic than lysosomes of untransformed mammary epithelial or benign breast cancer cells. Similarly, the lysosomes of pancreatic ductal adenocarcinoma (PDAC) cells with mutant KRAS show a >0.5 pH unit decrease compared to normal human pancreatic duct epithelial cells or PDAC cells lacking oncogenic KRAS. Transformation of normal kidney or mammary cells with mutant KRAS or mutant HRAS, respectively, results in a lysosome acidification similar to cancer cells. Interestingly, mutant RAS transformation-mediated reduction in lysosome pH coincides with cytosol alkalinization, but a direct link between these observations remains to be established. Nonetheless, a direct link between alkaline pHi and lysosome pH was found for cervical cancer cells, where loss of lysosome acidification coincides with a reduction in cytosolic pH, which points to lysosomes functioning as a proton sink (Liu et al., 2018). The lysosome as a proton sink in cancer is extensively reviewed by Chen et al. (2020). The *medial*/*trans*-Golgi is proposed to become more alkaline in cancer, as determined by comparing various human cancer cell lines to canine kidney epithelial cells and human and monkey fibroblasts (Rivinoja et al., 2006; Kokkonen et al., 2019; Khosrowabadi et al., 2021). However, no differences in luminal pH were observed when comparing the *trans*-Golgi network of Chinese hamster ovary (CHO) and cervical cancer cells (Demaurex et al., 1998). If there is indeed an increase in Golgi pH in cancer cells, this might not necessarily be linked to reduced proton loading since organelle pH also relies on buffering capacity. Such a buffering effect could result in increased luminal pH, while still allowing for the capture of protons from the cytosol. The alkalinization in the Golgi is proposed to occur through exchanger-mediated bicarbonate buffering that reduces the luminal proton content by converting HCO_3_^–^ and H^+^ into H_2_O and CO_2_, which can readily occur at the resting pH of the Golgi since the pKa of HCO_3_^–^ is 6.4 (Khosrowabadi et al., 2021). With this increased pH buffering capacity, the Golgi in cancer cells is possibly better equipped to tolerate proton sequestration and allow for additional proton loading relative to normal cells, which may contribute to clearing cytosolic protons. However, additional studies are required to confirm this potential.

## Golgi pH Regulation

The Golgi pH is thought to be regulated at multiple levels through proton pumping, proton leakage, buffering, and counter ion transport. Significant advances have been made in the understanding of these processes, but a comprehensive model for luminal Golgi pH regulation in cancer largely remains unresolved. Regulators of Golgi pH have mainly been identified in non-cancerous models and the variety of cancer types and their genomic differences may contribute to alternative regulation. Moreover, cancer frequently displays changes in ion channel expression levels and localization and is therefore proposed as a channelopathy, which, given the multitude of ion channels that can set off pH changes, beclouds the direct translation of non-cancerous findings to cancer models (Litan and Langhans, 2015; Prevarskaya et al., 2018). Notably, it should be considered that changes in expression levels do not necessarily translate to changes in pH levels since ion channel activity can also be driven by membrane potential and ion gradients. Here, we will outline the regulators of Golgi pH homeostasis thus far identified (Figure 1).

### Vacuolar H^+^-ATPases

The Vacuolar H^+^-ATPase (V-ATPases) is the main acidifier of organelles along the endocytic and secretory pathway and its activity is required for both endocytosis and exocytosis (Vasanthakumar and Rubinstein, 2020). The importance of the proton pump in Golgi acidification is well illustrated by studies from Llopis et al. (1998) who showed that V-ATPase inhibition with bafilomycin A1 led to Golgi alkalinization, reaching pH values close to the cytosolic pH levels. The proton pump is a multi-subunit protein complex that transports H^+^ ions across the membrane using ATP as an energy source. Expression levels of the different subunits are tissue-, cell-, and organelle-specific, and, in cancer, the subunits are frequently differentially expressed (Couto-Vieira et al., 2020). The localization of V-ATPase to the Golgi and endosomes is thought to occur through the V0a2 subunit, and its role in proper Golgi function is illustrated by the Golgi dysfunction observed in cutis laxa patients, which harbor a mutation in *ATP6V0A2* (Kornak et al., 2008; Hucthagowder et al., 2009). In cancer, V0a2 subunit expression is mostly unaffected at the transcriptional level (*ATP6V0A2*,^1^) (Tang et al., 2019), but the subunit may show differential localization to the plasma membrane, as observed in ovarian cancer (Kulshrestha et al., 2015). Here, knockdown of the subunit results in cytosolic acidification, indicating that V-ATPase activity is important for pHi homeostasis, but additional studies are required to determine if the Golgi is involved (Kulshrestha et al., 2016).

The V-ATPase proton pump has been demonstrated to transport protons constitutively into the Golgi lumen, where the resting pH is dictated by a balance between proton pumping and proton leakage (Schapiro and Grinstein, 2000; Wu et al., 2001). Inhibition of V-ATPases causes luminal alkalinization of the Golgi and secretory vesicles, highlighting the role for V-ATPase in acidifying these organelles. However, artificial luminal acidification in combination with V-ATPase inhibition only results in gradual alkalinization of the Golgi and is not detected in secretory vesicles (Wu et al., 2001). These results indicate the possible presence of a proton leak pathway at the Golgi, which is non-existent or minimal in secretory vesicles. Interestingly, at the lysosome, V-ATPase is shown to be regulated by the oncoprotein STAT3, which increases proton pump activity in response to sensing cytosolic acidification, thereby restoring the alkaline pHi in cancer (Liu et al., 2018). The role of STAT3 in regulating Golgi acidification seems to be absent, since the study did not find evidence for STAT3 localization at the Golgi. Nevertheless, a large number of proteins have been identified to interact with the V-ATPase proton pump, which could possibly affect its Golgi acidification capacity (Merkulova et al., 2015).

### Na^+^/H^+^ Exchangers

Sodium-hydrogen exchangers (NHEs) are a family of electroneutral membrane ion transporters that transfer protons across membranes in a 1:1 exchange for Na^+^, and in some cases Li^+^ and K^+^ (Pedersen and Counillon, 2019). The family consists of nine isoforms that share a conserved architecture and can be classified into two groups: the plasma membrane NHEs (NHE1-5) and the endomembrane NHEs (NHE6-9), although NHE8 can be considered a separate group as it exerts its function at both the plasma membrane and endomembranes. Both NHE7 and NHE8 have been identified to localize to the Golgi and have been implicated in the regulation of the Golgi resting pH (Numata and Orlowski, 2001; Nakamura et al., 2005). NHE7 expression is frequently increased in cancer (*SLC9A7*,^1^ (Tang et al., 2019) and overexpression of NHE7 in a breast cancer cell line enhances cell adhesion, invasion, and anchorage-independent growth (Onishi et al., 2012). No significant differences are observed for NHE8 expression in tumors relative to tissue-matched controls and no data is available for its role in cancer (*SLC9A8*,^1^) (Tang et al., 2019).

The NHE7 and NHE8 exchangers were originally proposed to function as a Golgi leak pathway for protons (Numata and Orlowski, 2001; Nakamura et al., 2005), allowing H^+^ to flow out of the lumen through the ion gradient formed by the elevated H^+^ levels relative to the cytosol. The notion that sodium-hydrogen exchangers act as a proton leak pathway was supported by the original findings that exogenous NHE8 expression in monkey fibroblasts increased the pH of the Golgi and NHE7 expression in CHO cells increased the Na^+^ influx into endomembrane structures (Numata and Orlowski, 2001; Nakamura et al., 2005). However, CHO cells lacking plasma membrane NHE1 did not confirm the NHE7 leak pathway since the Golgi resting pH was unaltered after exogenous NHE7 expression (Khayat et al., 2019). Nevertheless, exogenous expression of an NHE7 mutant linked to intellectual disability did cause alkalinization of the Golgi. According to the proton-leak hypothesis, the authors proposed a model in which the mutation transformed the exchanger into a hyperactive proton leak responsible for the luminal alkalization (Khayat et al., 2019). Subsequent studies on NHE8 have pointed toward a role in regulating the function and morphology of multivesicular bodies, with no observed changes in luminal pH (Lawrence et al., 2010). The proton-leak hypothesis warrants further scrutiny, especially since an increase of cytosolic Na^+^ to 103 and 140 mM, concentrations similar to physiological extracellular concentrations known to drive NHE1-mediated proton extrusion, had minimal effects on the Golgi pH, suggesting that the Na^+^/H^+^ leak activity at the Golgi is insignificant (Demaurex et al., 1998; Schapiro and Grinstein, 2000).

An opposing hypothesis for the function of NHE7 at the Golgi is in a role as a proton loader. NHE7 expression is frequently increased in cancer, as is the case for PDAC (Galenkamp et al., 2020). Here, endogenous NHE7 localization was confirmed at the *trans*-Golgi network and knockdown of NHE7 resulted in alkalization of the organelle and a concomitant increase in cytosolic pH. A thorough assessment of NHE7 features by Milosavljevic et al. (2014) revealed that the exchanger functions as an acid loader. Importantly, NHE7 was shown to display non-reversible proton transport from the cytosol to the lumen, arguing against NHE7 being able to function as a proton leak pathway at the Golgi. Moreover, the study indicated that NHE7-mediated proton loading is only effectuated by high Na^+^ concentrations, not by K^+^, and is constitutively activated by cytosolic H^+^.

### Anion Exchangers

The anion exchangers (AEs) family contains membrane transporters that electroneutrally and reversibly exchange Cl^–^ for HCO_3_^–^ (Romero et al., 2013). The exchangers mediate bicarbonate buffering that contributes to pH homeostasis by sequestering H^+^ at acidic pH levels and which leads to the conversion of protons and bicarbonate into water and CO_2._ Both products can readily escape the lumen of organelles through diffusion and, possibly, aquaporin water channels (Nozaki et al., 2008; Alberts, 2015). Of special interest is the Golgi-localized AE2 isoform AE2a (Holappa et al., 2001). AE2 gene transcription is upregulated in multiple cancer types and is linked to promoting cell viability, proliferation, migration and invasion of cancer cells (*SLC4A2*,^1^) (Hwang et al., 2009, 2019; Zhang et al., 2017; Celay et al., 2018; Tang et al., 2019; Khosrowabadi et al., 2021). The AE2a isoform is reported to increase the Golgi resting pH and therefore represents a likely candidate for the proton leak observed at the Golgi (Wu et al., 2001; Khosrowabadi et al., 2021).

### Golgi pH Regulator

The Golgi pH regulator (GPHR) is a voltage-gated chloride channel that regulates Golgi pH through counter ion conductance (Maeda et al., 2008). The influx of chloride ions into the Golgi allows for H^+^ pumping by reducing the membrane potential, which increases through continuous H^+^ pumping by the V-ATPases (Llopis et al., 1998; Maeda et al., 2008). Moreover, GPHR can conceivably provide the chloride ions required for anion exchanger-mediated bicarbonate transport at the Golgi (Becker and Deitmer, 2020). GPHR is mainly localized to the Golgi and the introduction of an inactivating mutation or downregulation increases the Golgi resting pH, causes disruption of Golgi integrity, impairs glycosylation, and Golgi to plasma membrane trafficking (Maeda et al., 2008; Vavassori et al., 2013; Sou et al., 2019). Little is known about the role of GPHR in cancer and its expression is largely unaffected by transformation (*GPR89A/B*,^1^) (Tang et al., 2019).

### Transmembrane Protein 165

The ion specificity of transmembrane protein 165 (TMEM165) is still debated, but research findings point to the function of a Ca^2+^/Mn^2+^ transporter in exchange for H^+^ (Lebredonchel et al., 2019; Stribny et al., 2020; Wang et al., 2020). TMEM165 localizes to the Golgi and knockdown of TMEM165 in normal liver cells results in Golgi acidification, suggesting TMEM165 is a proton leak pathway (Foulquier et al., 2012; Wang et al., 2020). However, in cervical cancer cells knockdown of TMEM165 was shown to cause acidification of lysosomes (Demaegd et al., 2013). Nonetheless, the role of TMEM165 as a possible regulator of Golgi pH is supported by glycosylation abnormalities found in patients harboring a *TMEM165* mutation, which is tightly linked to Golgi pH homeostasis (Foulquier et al., 2012). TMEM165 is upregulated in a few cancer types and is linked to promoting migration and invasion (*TMEM165*,^1^) (Lee et al., 2018; Tang et al., 2019; Murali et al., 2020).

### Cystic Fibrosis Transmembrane Conductance Regulator

The role of cystic fibrosis transmembrane conductance regulator (CFTR) in regulating Golgi pH is controversial since studies have described seemingly contradictory findings. Moreover, CFTR is not a Golgi-resident protein *per se* but traffics through the Golgi to reach the cell surface. Nonetheless, this counterion channel has been shown to change Golgi pH in a cystic fibrosis model (Poschet et al., 2001). CFTR is a cAMP-activated Cl^–^/HCO_3_^–^ channel best known for causing the life-limiting cystic fibrosis disease through the F508del mutation, but mutations and differential expression are also linked to cancer predisposition (Amaral et al., 2020). Gene expression is increased or reduced depending on the cancer type (*CFTR*,^1^) (Tang et al., 2019). The F508del mutation causes CFTR misfolding, ER retention, and degradation (Cheng et al., 1990; Denning et al., 1992). In a PDAC cell line derived from a cystic fibrosis patient, the F508del mutation was shown to cause Golgi dispersion which was reverted by wild-type CFTR expression (Hollande et al., 2005). Cells harboring the mutation show hyperacidification of the Golgi, which is counteracted by restoring Δ508-CFTR folding or reintroduction of wild-type CFTR (Chandy et al., 2001; Poschet et al., 2001). However, reduced Cl^–^ influx by decreased expression of CFTR at the Golgi cannot explain the increased acidity, since Cl^–^ is a H^+^ counterion that reduces the membrane potential. This led to the proposal of a model in which Na^+^ efflux from the organelle is increased in the absence of CFTR, allowing for additional H^+^ pumping (Poschet et al., 2002). The CFTR chloride channel represses sodium efflux by inhibiting the epithelial sodium channel, ENaC, resulting in Na^+^ build up due to the action of Na^+^/K^+^-ATPases. This leads to reduced proton pumping in response to the increase in membrane potential caused by Na^+^ ions. Nonetheless, the role of CFTR in regulating Golgi pH remains controversial as administration of cAMP had little effect on Golgi pH of CFTR mutant and wild-type cells (Llopis et al., 1998; Chandy et al., 2001). This in contrast to previous studies where cAMP was found to alkalinize the Golgi, but where overexpression of CFTR did not significantly change the pH of the organelle (Seksek et al., 1995, 1996).

## Discussion

The concept of the Golgi as a proton sink remains to be fully explored and requires more extensive studies that specifically address the contribution of the Golgi to maintenance of cytosolic pH. Moreover, plasma membrane transporters are historically thought to predominately regulate pHi and the role of organelles in this homeostatic process needs additional scrutinization (Paroutis et al., 2004; Casey et al., 2010; Webb et al., 2011; Corbet and Feron, 2017; Zheng et al., 2020). It would be interesting to examine the level of contribution that alternative pathways have on H^+^ efflux, such as exocytosis and proton loading of lysosomes and the Golgi, and whether these pathways’ contributions are distinctive for cancer vs. untransformed cells. In cancer, both the perturbation of Golgi and lysosome pH has been shown to affect the pH of the cytosol, but equivalent analyses of normal cells is lacking (Liu et al., 2018; Funato et al., 2020; Galenkamp et al., 2020). As an acidic organelle, the Golgi subtracts protons from the cytosol and, as a part of the secretory pathway, might target H^+^ for the extracellular space. Conceptually, it may be possible that the Golgi can contribute to pHi homeostasis in both cancer and normal cells. However, one observation that this might not be the case is that the depletion of NHE7 in normal pancreatic cells did not affect the cytosolic pH, while loss of NHE7 in PDAC cells resulted in alkalinization of the Golgi that caused a decrease in cytosolic pH (Galenkamp et al., 2020). A likely explanation is that organelle acidification plays a greater role in cancer cells due to the acidic burden that these cells have to withstand in response to amplified aerobic glycolysis and ATP hydrolysis (Webb et al., 2011; Corbet and Feron, 2017; Zheng et al., 2020). Alternatively, acidic organelles in cancer may exhibit increased or altered activity that exacerbates the effect on cytosolic pH when perturbed. Indeed, cancer cells show increased Golgi-mediated secretion which would increase the number of protons secreted through this pathway (Halberg et al., 2016; Capaci et al., 2020; Gupta et al., 2020; Tan et al., 2020). Given this enhanced secretory flux, perturbation of the secretory pathway might result in greater accumulation of protons in the cytosol, relative to untransformed cells. Additionally, cancer cells display altered luminal ion levels, and differential transporter levels or activity, which could potentially bring about changes in proton loading (Litan and Langhans, 2015; Prevarskaya et al., 2018).

The contribution of additional Golgi proton loaders or leak pathways and their physiological relevance alongside V-ATPase, which is the main acidifier of secretory and endocytic organelles, in cancer and normal cells is limitedly studied. In cancer, the Golgi is proposed to display elevated buffering capacity *via* increased AE2a expression and bicarbonate loading (Khosrowabadi et al., 2021). A conceivable source of bicarbonate for transport into the Golgi lumen are the carbonic anhydrases present at the cell surface, which are upregulated in cancer and convert carbon dioxide and water into H^+^ and HCO_3_^–^ (Mboge et al., 2018). In turn, bicarbonate is imported by the cell through Na^+^/HCO_3_^–^ cotransporters that utilize the existing Na^+^ gradient between the cytosol and extracellular fluid (Becker and Deitmer, 2020). Additionally, the cytosolic carbonic anhydrase CAII may provide HCO_3_^–^ as it has been determined to localize to the Golgi (Alvarez et al., 2001). Importantly, CAII and AE2 are able to form a transport metabolon, a complex between the anion exchanger and carbonic anhydrase, which is proposed to be required for full bicarbonate transport activity by AE2 (Vince and Reithmeier, 2000; Sterling et al., 2001; Gonzalez-Begne et al., 2007; Becker and Deitmer, 2020). Luminal chloride ions required for the counter ion transport in the HCO_3_^–^ exchange are most likely transported to the lumen through chloride channels, such as GPHR, present at the Golgi (Maeda et al., 2008). Although AE2a does not directly mediate proton leakage, but provides a means for proton neutralization, it presents a plausible option for the proton leak pathway that had previously been identified, but for which thus far a compelling candidate is lacking (Wu et al., 2001). Given that the pKa of bicarbonate is 6.4, in the Golgi lumen, bicarbonate and H^+^ can convert to water and carbon dioxide without requiring carbonic anhydrases (Mboge et al., 2018). This alkalinization of the Golgi by increased bicarbonate buffering may provide means for additional subtraction of protons from the cytosol. The contribution of the pathway to cytosolic pH alkalinization in cancer ultimately depends on the fate of the carbon dioxide and whether it remains within the cell and converts back to bicarbonate in a carbonic anhydrase-dependent or independent manner, or whether it leaves the cell and thereby leads to the neutralization of intracellular protons.

The sodium-hydrogen exchanger NHE7 had previously been proposed as a proton leak pathway at the Golgi, but more recent data has pointed toward NHE7 functioning as a proton loader (Numata and Orlowski, 2001; Milosavljevic et al., 2014; Galenkamp et al., 2020). Endogenous NHE7 localizes to the *trans*-Golgi network; however, the transporter can be present on post-Golgi vesicles, endosomes, and traffic to the plasma membrane when overexpressed or when endocytosis is inhibited (Numata and Orlowski, 2001; Lin et al., 2005, 2007; Nakamura et al., 2005; Fukura et al., 2010; Onishi et al., 2012; Khayat et al., 2019; Galenkamp et al., 2020; Lopez-Hernandez et al., 2020). Whether NHE7 is required for the formation of post-Golgi vesicles and endosomes, or whether it is a passenger, and if it regulates post-Golgi vesicle acidification has not been carefully assessed. When forced to express at the plasma membrane, NHE7 is shown to function as a proton loader of endosomes, with no leak activity (Milosavljevic et al., 2014). However, cells with and without NHE7 expression displayed similar steady-state endosomal pH levels. Inhibition of NHE7 with the pan-NHE inhibitor EIPA reduced acidification of endosomes to a similar extent as bafilomycin A1, whereas the effect of EIPA was absent in cells without NHE7 expression. These data indicate that NHE7 can function alongside V-ATPases in mediating luminal acidification and that the steady-state pH might involve both transporters. NHE7 knockdown in PDAC cells was shown to alkalinize the Golgi, but a direct evaluation of Na^+^-mediated proton loading of the Golgi lumen remains to be carried out (Galenkamp et al., 2020). Nonetheless, the involvement of NHEs in Golgi acidification in PDAC was confirmed by treatment with EIPA. Altogether, the data fits a model in which NHE7 acidifies the *trans*-Golgi network, possibly alongside V-ATPases. It cannot be completely ruled out that the downregulation or inhibition of NHE7 affects V-ATPase activity or that proton extrusion at the plasma membrane partially contributes to the observed effects. NHE7 constitutively binds protons (Km = 2.5 10^–7^ M), but has a low affinity for Na^+^ (Km = 240 mM) and thus needs high Na^+^ concentrations to drive proton transport against the gradient between the Golgi lumen and the cytosol (Milosavljevic et al., 2014). These findings suggest that, if NHE7 functions as an acid loader at the Golgi, a source of luminal Na^+^ should be present. Thus far the concentrations of Na^+^ at the Golgi have not been determined and might be differentially regulated in cancer. Na^+^/K^+^-ATPases present at the Golgi are likely not the source of Na^+^, since their inhibition did not affect Golgi pH in cervical cancer cells (Llopis et al., 1998) and reduced Golgi resting pH in cystic fibrosis control cells (Poschet et al., 2001). However, luminal Na^+^ concentration could be driven by retrograde transport from endosomes to the *trans*-Golgi network, which delivers extracellular Na^+^ obtained through endocytosis (Tu et al., 2020). This process is thought to contribute to the high Na^+^ concentrations observed in lysosomes, where Na^+^ is the predominant cation at a concentration of ∼150 mM (Wang et al., 2012; Xu and Ren, 2015). A role for this pathway in Na^+^ delivery to the Golgi has yet to be assessed.

Altogether, the available data indicates that additional transporters besides V-ATPase can co-regulate the luminal pH of the Golgi and contribute to the extraction of protons from the cytosol. The role of each of these transporters and whether the correct physiological conditions are met to allow for each of these transporters to function remains to be determined on a contextual basis and could be differentially regulated in untransformed cells vs. cancer.

## Conclusion

Cancer is a pathological state in which cells are exposed to chronic stresses that can alter cellular processes in order to provide growth benefits despite the harsh tumor microenvironment (Webb et al., 2011; Corbet and Feron, 2017; Zheng et al., 2020). One such stress is the increased production of H^+^ ions, which can lead to cellular acidification and tumor acidosis. Despite these conditions, cancer cells maintain an alkaline pHi that allows proliferation to thrive. The Golgi is shown to contribute to the upkeep of this alkaline cytosolic pH in cancer by functioning as a proton sink (Galenkamp et al., 2020). Whether the Golgi also plays a role in the maintenance of cytosolic pH in normal cells remains to be determined. In addition to the proton storage capacity, the Golgi contains inherent properties, the secretory pathway, to target protons for the extracellular space. This pathway becomes malignant in cancer and may promote proton extrusion and provide means to drive metastasis (Halberg et al., 2016; Capaci et al., 2020; Gupta et al., 2020; Tan et al., 2020). The limited understanding of the players involved in Golgi pH homeostasis in cancer impedes the targeting of this pathway as a therapeutic strategy. Further investigation is warranted to fully comprehend the contribution of the Golgi ion channels to Golgi pH and pHi homeostasis in cancer. It would be beneficial to scrutinize how ion channels in the Golgi are regulated, as they may be affected by changes in expression levels or subcellular localization, or through activation cascades, such as observed in the WNK signaling pathway, and by secondary messengers, such a cAMP and PDGF (Seksek et al., 1995; Alessi et al., 2014). Such examinations may provide valuable insights into ways to therapeutically disrupt pH homeostasis in cancer and may open new avenues for pharmacological intervention, which are eagerly needed to improve clinical outcomes for cancer patients.

## Author Contributions

Both authors conceived, organized, and wrote the manuscript.

## Conflict of Interest

The authors declare that the research was conducted in the absence of any commercial or financial relationships that could be construed as a potential conflict of interest.

## Footnotes

1 http://gepia2.cancer-pku.cn
